# Novel *KIF26A* variants associated with pediatric intestinal pseudo‐obstruction (PIPO) and brain developmental defects

**DOI:** 10.1111/cge.14621

**Published:** 2024-09-21

**Authors:** Mohammad Sadegh Shams Nosrati, Alireza Doustmohammadi, Mariasavina Severino, Ferruccio Romano, Mahdi Zafari, Amir Hesam Nemati, Clara Velmans, Christian Netzer, Jonas Breuer, Ilse Julia Broekaert, Alexander Joachim, Nihad Almasri, Michael C. Kruer, Peter Skidmore, Pritha Bisarad, Jumana Hoque, Somayeh Bakhtiari, Annalaura Torella, Vincenzo Nigro, Francesca Buffelli, Ezio Fulcheri, Annette Müller, Federico Zara, Valeria Capra, Marcello Scala

**Affiliations:** ^1^ Department of Neurosciences, Rehabilitation, Ophthalmology, Genetics, Maternal and Child Health University of Genoa Genoa Italy; ^2^ U.O.C. Genetica Medica IRCCS Istituto Giannina Gaslini Genoa Italy; ^3^ Department of Bioinformatics and Computational Biophysics, Faculty of Biology University of Duisburg‐Essen Essen Germany; ^4^ Neuroradiology Unit IRCCS Istituto Giannina Gaslini Genoa Italy; ^5^ Genomics and Clinical Genetics Unit IRCCS Istituto Giannina Gaslini Genoa Italy; ^6^ Department of Bioengineering Northeastern University Boston Massachusetts USA; ^7^ Department of Epidemiology and Biostatistics Pasteur Institute of Iran Tehran Iran; ^8^ Institute of Human Genetics, Faculty of Medicine and University Hospital Cologne University of Cologne Cologne Germany; ^9^ Department of Paediatrics, Faculty of Medicine and University Hospital Cologne University of Cologne Cologne Germany; ^10^ Department of Pediatrics, Faculty of Health Helios University Medical Center Wuppertal, Witten/Herdecke University Witten Germany; ^11^ Department of Rehabilitation Sciences College of Health Sciences Qatar University Doha Qatar; ^12^ Department of Physiotherapy School of Rehabilitation Sciences University of Jordan Amman Jordan; ^13^ Pediatric Movement Disorders Program, Division of Pediatric Neurology Barrow Neurological Institute, Phoenix Children's Hospital Phoenix Arizona USA; ^14^ Departments of Child Health, Neurology, and Cellular & Molecular Medicine, and Program in Genetics University of Arizona College of Medicine–Phoenix Phoenix Arizona USA; ^15^ College of Health Solutions Arizona State University Tempe Arizona USA; ^16^ Department of Precision Medicine University of Campania "Luigi Vanvitelli" Naples Italy; ^17^ Telethon Institute of Genetics and Medicine Pozzuoli Italy; ^18^ Fetal‐Perinatal Pathology Unit IRCCS‐Istituto Giannina Gaslini Genoa Italy; ^19^ Pediatric Pathology University Clinic of Cologne Cologne Germany

**Keywords:** brain malformations, congenital megacolon, exome sequencing, *KIF26A*, kinesin, neurodevelopmental disorder

## Abstract

Pediatric intestinal pseudo‐obstruction (PIPO) is a rare congenital disorder of the enteric nervous system with distal colon aganglionosis potentially leading to intestinal obstruction. Recently, biallelic variants in *KIF26A*, encoding a crucial motor protein for the migration and differentiation of enteric neural crest cells, have been associated with a neurodevelopmental condition featuring cortical defects and PIPO‐like features, though in absence of aganglionosis. So far, only 10 patients have been reported. In this study, we investigated three subjects with congenital hydrocephalus, neurodevelopmental impairment, and intestinal obstruction megacolon syndrome. Brain MRI revealed malformations within cortical dysplasia spectrum, including polymicrogyria and heterotopia. Pathology study of the intestine revealed aganglionosis and elevated acetylcholinesterase activity in parasympathetic nerve fibers. Through trio‐exome sequencing (ES), we detected four novel biallelic *KIF26A* variants, including two missense changes (#1) and two distinct homozygous truncating variants in (#2 and #3). All variants are rare and predicted to be deleterious according to in silico tools. To characterize the impact of the missense variants, we performed 3D protein modeling using Alphafold3 and YASARA. Mutants exhibited increased energy scores compared to wild‐type protein, supporting a significant structural destabilization of the protein. Our study expands the genotype and phenotype spectrum of the emerging *KIF26A*‐related disorder.

## Graphical abstract

This study identifies novel biallelic KIF26A variants in three patients with congenital hydrocephalus and intestinal obstruction. Using 3D protein modeling, we reveal that missense variants cause significant structural destabilization of the KIF26A protein. Additionally, histopathology data show aganglionosis and elevated acetylcholinesterase activity, expanding the genotype–phenotype spectrum of this emerging disorder.

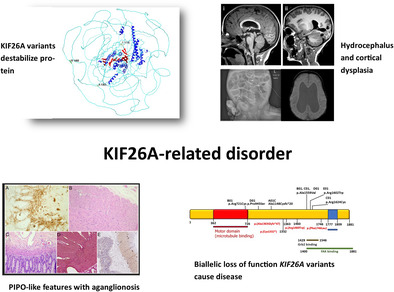

## 1 INTRODUCTION

Pediatric intestinal pseudo‐obstruction (PIPO) is defined as a chronic motility disorder of the gastrointestinal tract mimicking mechanical obstruction.^1^ The diagnosis of PIPO requires the absence of ganglion cells in the colon, resulting in functional bowel obstruction.^2^ the development of the enteric nervous system (ENS) relies on a complex interplay of genetic and acquired/environmental factors.^2^ These conditions can occur as manifestations isolated or in association with other symptoms in complex syndromes.^2^ many different genes have been involved in the pathogenesis of ENS disorders, recent research draws attention to the association between *KIF26A* and PIPO. The *KIF26A* gene (MIM*613231) encodes a motor protein pivotal for intracellular transport and microtubule dynamics and playing a significant role in the migration and differentiation of enteric neural crest cells.^3^ In mice, *Kif26a* knockout (KO) results in megacolon, enteric neuronal hyperplasia, and impaired neurite outgrowth.^4^

Recently, biallelic *KIF26A* variants have been associated with a complex condition characterized by brain malformations and megacolon, suggesting a potential link between KIF26A dysfunction and of PIPO.^5^, ^6^

So far, only 10 patients from 7 unrelated families have been reported in the literature and many died during infancy, resulting in a poor delineation of the disease course (see Table 1 for a summary of the genetic and clinical features of KIF26A patients). Patients harboring *KIF26A* variants show dysmorphism, psychomotor impairment, and a spectrum of brain developmental defects featuring polymicrogyria, agenesis of the corpus callosum, and ventriculomegaly.^6^ Experiments based on animal and human brain organoid models showed, that *KIF26A* plays a crucial role in brain development as a regulator of intracellular signaling pathways overseeing radial migration, neurites and axons growth, and regulation of apoptosis in cortical excitatory neurons during normal brain development and function.^6^

**TABLE 1:** Summary of the genetic and clinical features of *KIF26A* patients.

	Case 1	Case 2	Case 3	#1 PMID: 36064622	#2 PMID: 36064622	#3 PMID: 36064622	#4 PMID: 36064622	#5 PMID: 36064622	# 1PMID: 36228617	#2 PMID: 36228617	#3 PMID: 36228617	#4 PMID: 36228617		#5 PMID: 36228617
	8 years, M	6 months, F	8 years, F	14 months, F	3.5 years, M	4 months, M	2 months, M	2 months, M	21 gestational weeks, M	3 month, M	17 years, M	30, M		3 years 6 months?, M
*KIF26A* variant (NM_015656.1)	c.4378C > T, p.(Arg1460Trp); c.5238C > G, p.(Phe1746Leu)	c.4085dup, p.(Ala1363Glyfs*47)	c.3996C > A, p.(Cys1332*)	c.792dupC, p.(Val265Argfs*5)	c.792dupC, p.(Val265Argfs*5)	?	c.792dupC, p.(Val265Argfs*5)	c.3330delC, p.(Ser1111Alafs*137)	c.2161C > T, p.(Arg721Cys); c.4676C > T, p.(Ala1559Val)	c.3440dupC, p.(Ala1148Cysfs*20)	c.4676C > T, p.(Arg1624Cys); c.4870C > T, p.(Ala1559Val)	c.2845C > T, p.(Pro949Ser); c.4676C > T, p.(Ala1559Val)		c.4804C > T, p.(Arg1602Trp)
Psychomot or delay	+	+	+	NA	+	NA	NA	+	NA	NA	NA	NA		NA
Motor delay	+	NA	+	NA	+	NA	NA	+	NA	NA	NA	NA		NA
Speech delay	−	NA	+	NA	+	NA	NA	+	NA	NA	NA	NA		NA
Intellectual disability	−	NA	+	NA	NA	NA	NA	NA	NA	NA	+	NA		NA
DD/ID severity	Mild	−	−	Mild	Moderate	Moderate	NA	NA	NA	NA	Mild	NA		+
Facial dysmorphism	+	−	−	−	NA	NA	−	−	−	−	−	NA		NA
Abnormal OFC	−	+	−	−	−	−	+	−	+ (microcephaly)	−	−	NA		NA
Neurologic al features														
Hypotonia	+	+	−	−	NA	NA	NA	NA	NA	NA	NA	NA		NA
Spasticity	−	−	+	−	NA	NA	NA	+	NA	NA	NA	NA		NA
Dysarthria	−	−	−	NA	NA	NA	NA	NA	NA	NA	NA	NA		NA
Epilepsy	−	−	+Receiving	−	−	−	−	−	−	−	−	−		−
Seizure onset	−	−	treatment	−	−	−	−	−	−	−	−	−		−
Seizure type	−	−	−	−	−	−	−	−	−	−	−	−		−
Response to AEDs	NA	NA	− NA	−	−	NA	NA	NA	NA	NA	NA	NA		NA
Hirschsprung disease/PIPO	+	+	−	−	−	−	+	−	−	−	−	−		−
Cardiac defects	+	+	−	+	+	+	+	NA	NA	NA	NA	NA		NA
Other clinical manifestations	Dacryocystocele, dyscrania, clinodactyly	Arachnodactyly	Hearing problems	Dehydration, fever	Ischemic small bowels	Ascites	−	Intestinal obstruction	Megacolon	High arched palate and long philtrum	High arched palate and long philtrum	−		−
Brain MRI														
Ventriculomegaly/Hydrocephalus	+	+	NA	+	+	+	+	+	+	+	+	+		+
Gyration defects	+	−	NA	−	−	−	−	−	−	−	−	+		+
Heterotopia	+	−	NA	−	−	−	−	−	−	−	−	−		−
CCH/CCA	−	+	NA	+	+	−	−	−	+	+	+	−		+
Other malformations	+	Hypogenesis of septum pellucidum	NA	Brainstem patterning disorder	Brainstem patterning disorder	Long midbrain and small pons		CC squaring, long midbrain	Brain atrophy, reduced white matter	Bilateral schizencephaly	Absent hippocampal commissure, reduced white matter, schizencephaly	−	−	−

Abbreviations: CC, corpus callosum; CCA, corpus callosum agenesis; CCH, corpus callosum hypoplasia; PIPO, intestinal pseudo‐obstruction.

In this study, we describe three novel unrelated patients harboring biallelic variants in *KIF26A* and presenting with neurodevelopmental disorder, congenital megacolon with features of Hirschsprung's disease, and brain defects. Our findings further support the relevance of *KIF26A* in the etiology of ENS anomalies and brain development defects, expanding the genotype and phenotype of the emerging KIF26A‐related disorder (Table 1).

## 2 CASE PRESENTATION

This study adheres to the Declaration of Helsinki principles. The following Research Ethics Committees approved it: Gaslini Children's Hospital (Comitato Etico della Regione Liguria (163/2018). The authors obtained and archived written informed consents from parents or legal guardians of enrolled subjects to publish genetic and clinical data, including clinical photographs and brain MRI images.

### 2.1 Case #1

**FIGURE 1:**
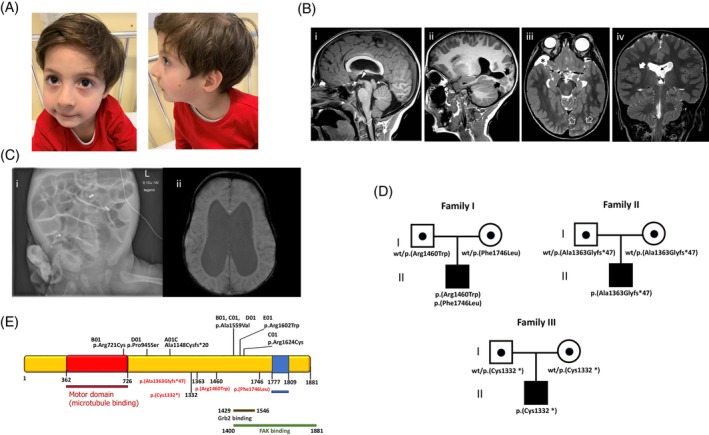
Clinical and genetic features of KIF26 patient. (A) Facial dysmorphism includes sparse eyebrows, almond‐shaped eyes, flat nasal bridge, thin lips, left mandibular hypoplasia with retrognathia, left‐sided helix hypoplasia, and bilateral lobe hypoplasia. (B) Brain MRI of the patient 1# performed at 6.5 years of age; (Bi–ii) Sagittal T1‐weighted images demonstrate cerebral aqueduct stenosis (curved arrow) associated with a small anterior commissure (dashed arrow) and hypothalamic adhesion (full arrowhead). There are several nodules of gray matter heterotopia extending from the parieto‐occipital cortex to the ventricle (thick black arrows). Note the caudal displacement of the cerebellar tonsils (empty arrowhead). (Biii) Axial T2‐weighted image shows bilateral incomplete hippocampal rotation. Note that the cortex overlying the nodular heterotopias is abnormal, showing a focal area of polymicrogyria (empty white arrows). (Biv) coronal T2‐weighted image depict enlargement of the lateral ventricles associated with focal fenestration of the septum pellucidum (thin black arrow). There is an additional area of polymicrogyria in the left insula (thick white arrow). (C) Brain MRI in patient #2: Hydrocephalus with obliterated aqueduct, hypoplasia of corpus callosum, agenesis of septum pellucidum. (Ci) x‐say: Intestinal dilatation due to distal obstruction. (Cii) Brain MRI: Dilatation of lateral. (D) Pedigree of the families showing the segregation of *KIF26A* variants in the proband and healthy parents. (E) Localization of previously reported (black) and novel (red) *KIF26* variant in the protein.

An 8‐year‐old boy of Italian ancestry was born via C‐section at 38 + 2 weeks. Birth measurements were within the 31st percentile, and echocardiogram and renal ultrasound results were normal except for a mild patent foramen ovale. Physical examination showed axial hypotonia and poor suction and failed to pass meconium, leading to surgery for Hirschsprung's disease. Post‐surgery, he had decreased stool frequency managed with macrogol. He was diagnosed with developmental delay, with a psychomotor assessment at age 4 showing mild right‐sided hypofunction. Physical examination revealed mild dyscrania, facial dysmorphisms (Figure 1A). Additional findings included a simian crease on the left hand, low‐placed fifth toe, and clinodactyly of the fourth toe on the right. The patient had narrow shoulders and walked with valgus feet. He could walk only when assisted.

### 2.2 Case #2

A 6‐month‐old girl from Syria was, born at 38 + 6 weeks by C‐section. Birth parameters were normal except head circumference (38 cm, >99th percentile). She presented with abdominal distension and intestinal obstruction symptoms. Imaging, including ultrasonography, x‐ray, and MRI, revealed dilated bowels without mechanical obstruction (Figure 1C). Diagnostic laparoscopy was performed for persistent vomiting, ileus‐like symptoms, and dependence on parenteral nutrition. Physical examination showed arachnodactyly, persistent foramen ovale, mild aortic valve stenosis, hypotonia, and psychomotor delay. She experienced multiple infections, including catheter‐related sepsis, CMV hepatitis, and colitis. During follow‐up, she continued to suffer ileus‐like symptoms, vomiting, long‐term parenteral nutrition dependence, recurrent infections, unstable blood electrolytes, and ileostomy prolapse. The patient received an ileostomy and enteral nutrition could be established.

### 2.3 Case #3

This patient is a girl born to consanguineous parents of Palestinian and Jordanian ancestry. She was delivered at 38 weeks gestation and birth parameters were within normal ranges. After birth, she was admitted to the neonatal intensive care unit and later diagnosed with spastic quadriplegic cerebral palsy (CP), characterized by severe motor delays (GMFCS Level V) and notable speech impairments (communication classification level III). Follow‐up clinical evaluations revealed a psychomotor developmental delay. The patient was unable to walk and could only crawl for short distances. She also experienced epileptic episodes. Additionally, she was diagnosed with hearing problems and cognitive impairments.

## 3 NEUROIMAGING STUDY

Brain MRIs were available for review in patients #1 and #2.

In patient #1, brain MRI at the age of 2 months revealed focal areas of polymicrogyria in the posterior right insula and left occipital lobe associated with occipital periventricular and transmantle nodular heterotopia. There was narrowing of the cerebral aqueduct with dilated lateral ventricles, low‐lying fornices, and septum pellucidum fenestration. The anterior commissure was small. There was mild pontine and inferior vermis hypoplasia associated with cerebellar foliar anomalies and mild caudal displacement of the cerebellar tonsils (<5 mm). Incomplete bilateral hippocampal rotation, hypothalamic adhesion and a small right temporal arachnoid cyst were also noted. Follow‐up brain MRI at 4 and 6.5 years of age demonstrated unchanged ventricular dilatation and increase in size of the arachnoid cyst (Figure 1B).

In patient #2, brain MRI confirmed the presence of the hydrocephalus diagnosed during pregnancy, associated with obliterated aqueduct, corpus callosum hypoplasia, and septum pellucidum agenesis (Figure 1C).

## 4 EXOME SEQUENCING

Exome sequencing (ES) was performed on genomic DNA extracted from peripheral blood of the patients and their parents.^7^ Candidate variants were assessed based on allele frequency, residue conservation, and predicted protein impact, as previously described.^8^ Then, ACMG criteria were used for variant classification. ES identified compound heterozygous variants in the *KIF26A* gene (NM_015656, NP_056471.1) in patient #1: the maternally c.4378C > T; p.(Arg1460Trp) in exon 12 and the paternally c.5238C > G; p.(Phe1746Leu) in exon 13 (Figure 1D,E). Both variants are rare in the general population (gnomAD v4.0.0; allele frequency 0.0000429 and 0.0000184, respectively); affect conserved residues, and are classified as variants of uncertain significance (VUS) (Table S1). patient #2 harbored the homozygous truncating variant c.4085dup; p.(Ala1363Glyfs*47), while Patient #3 carried the homozygous stop‐gain variant c.3996C > A; p.(Cys1332*). Both these variants are very rare, with an allele frequency 0.00000865 of 0.00000412 respectively, are predicted to be highly deleterious, and are classified as pathogenic (see Supplementary Materials for Table S1 on Summary of genetic variants description).

## 5 HISTOPATHOLOGICAL EXAMINATION

Pathology data were available for review in subjects #1 and #2.

**FIGURE 2:**
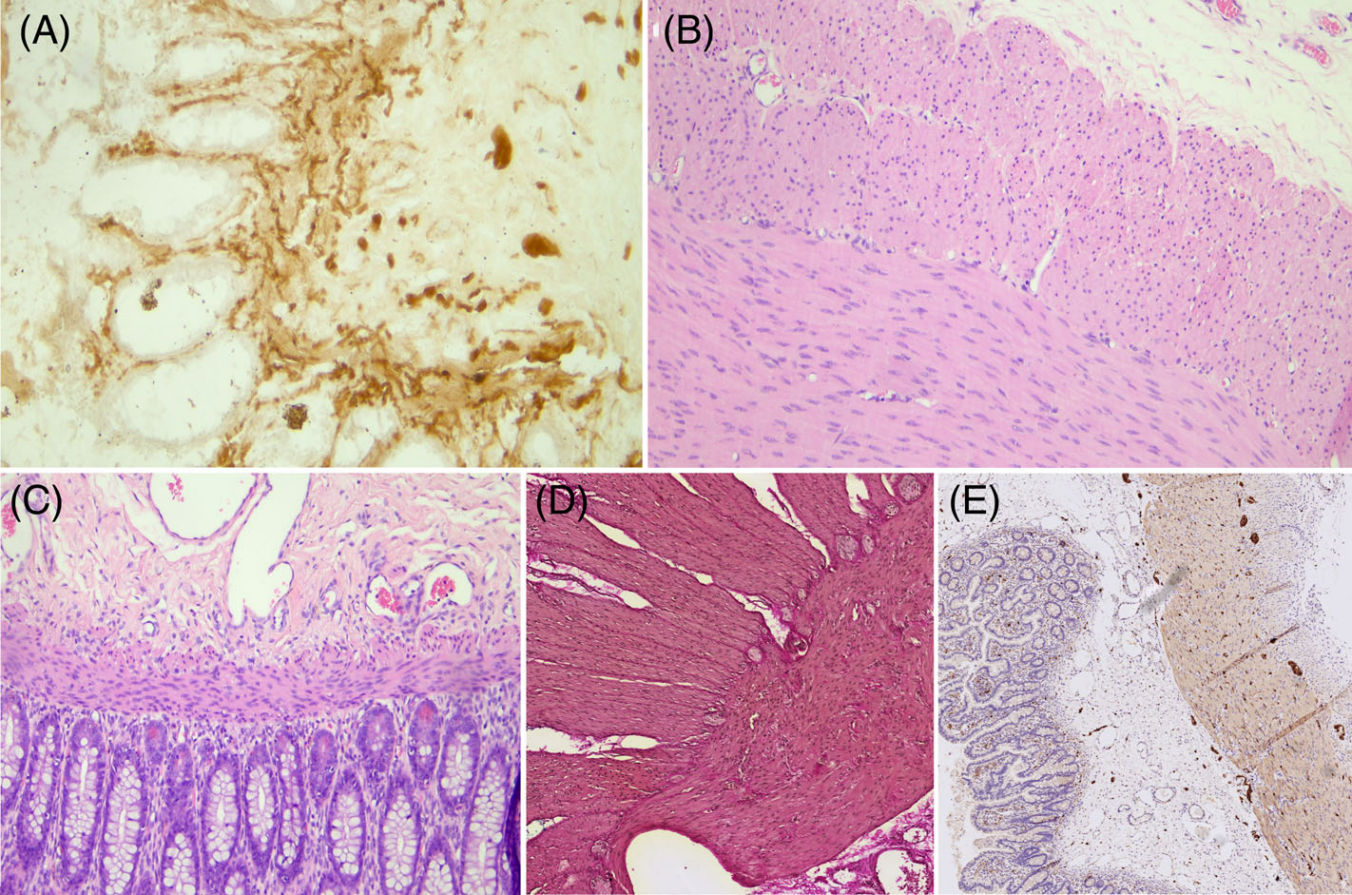
Histopathological examination. Patient 1# (A) Acetylcholinesterase: Initial biopsy comprising rectal mucosa and submucosa where an increase in acetylcholinesterase activity is recognized in the parasympathetic nerve fibers, ganglion cells are not recognizable. (B) Hematoxylin Eosin: Intestinal resection demonstrating a segment with aganglionosis at the level of the myenteric plexus. (C) Hematoxylin Eosin: Intestinal resection demonstrating a segment with aganglionosis at the level of the submucosal plexus. Patient 2#. (D) Elastica‐van‐Gieson staining: Biopsies of the small and large intestine showing ganglia, indicative of hypoganglionosis. (E) S100‐staining: Second biopsy confirming the diagnosis of hypoganglionosis, characterized by increased distances between ganglia.

In Case #1, histopathological examination on surgical samples revealed features consistent with PIPO diagnosis (Figure 2A–C). There were an increase in acetylcholinesterase activity in the parasympathetic nerve fibers, a segment with aganglionosis in the myenteric plexus, and a segment with aganglionosis in the submucosal plexus.

In Case #2, Biopsies of small and large intestine showed hypoganglionosis, defined as the presence of distances between ganglia greater than 2 ganglia in diameter (Figure 2D,E).

## 6 PROTEIN MODELING

To assess the impact of missense variants in patient #1, we predicted the 3D structure of the wild‐type (WT) protein using AlphaFold3 (Figure S1). Then, we evaluated the structural effects through AlphaFold3 for direct mutation modeling and YASARA to replace WT amino acids with the mutated ones. Energy levels were calculated using Rosetta 3.14, and structures were visualized with UCSF ChimeraX (Figures S1–S4). Energy scores (Table S2) showed the WT protein at 12285.839 REU as the most stable conformation. The missense variants had high scores (15742.053 REU and 13403.687 REU, respectively) indicating destabilization, as also supported by the 9% and 28% increase in energy (see Supplementary Materials for detailed methods, tables, and visualizations).

## 7 DISCUSSION

Deleterious variants in several genes involved in the development of the ENS have been suggested to contribute to Hirschsprung disease.^2^ Deleterious variants in the *RET* (MIM * 164761) disrupting the normal development of enteric nerve cells may lead to segmental aganglionosis^9^ and variants in other genes, such as *EDNRB*, *GDNF*, *GFRA1*, *KIF26A*, *PHOX2B* and *SOX10*, have been also suggested to contribute.^2^, ^10^, ^11^, ^12^ In particular, KIF26A dysfunction has been associated with ENS developmental defects.^5^, ^6^ This kinesin is crucial for the development and function of the ENS via the GDNF‐Ret pathway.^6^ It is a unique member of the Kinesin‐11 family attaching to microtubules but unable to perform ATP hydrolysis.^4^ In mouse enteric neurons, Kif26a interacts with Grb2, disrupting the Grb2/SHC complex formation, thereby inhibiting RET tyrosine kinase activation and subsequent signaling through the Ras‐MAPK and PI3K‐Akt pathways.^4^, ^13^, ^16^ KIF26A recruits focal adhesion kinase (FAK) to cytoplasmic microtubules, preventing FAK phosphorylation by Src family kinases and stabilizes microtubules; its absence disrupts microtubule dynamics.^14^, ^17^, ^18^, ^19^ In humans, KIF26A deficiency mirrors the intestinal phenotype seen in Kif26a−/− mice, marked by failure to establish normal neuronal networks despite myenteric neuronal hyperplasia.^5^ This results in megacolon resembling Hirschsprung's disease without aganglionosis.^5^, ^6^

In this study, we report three novel patients showing defects in central and enteric nervous systems. The p.(Arg1460Trp) and p.(Phe1746Leu) variants identified in patient #1 affect highly conserved residues critical for binding to GRB2 and FAK, which are involved in regulating radial neuronal migration and PI3K/AKT/MAPK signaling. 3D protein modeling revealed that these variants cause a significant structural destabilization in the protein, likely disrupting the interactions with GRB2 and FAK. The homozygous truncating variants p.(Ala1363Glyfs*47) and p.(Cys1332*) detected in patients #2 and #3 are predicted to lead to complete loss of function due to the generation of a nonfunctional truncated transcript or, alternatively, to nonsense‐mediated decay (NMD).The clinical manifestations of our patients align with the phenotypic spectrum of *KIF26A*‐related disorder.^5^

However, we observed severe spastic quadriplegic cerebral palsy (CP) with epilepsy in patient #3, suggesting that the neurological involvement in KIF26A patients may be heterogeneous. The lack of intestinal pathology data in previous reports makes it difficult to estimate the penetrance of ENS dysfunction in this condition.^5^, ^18^, ^19^ Among previous cases, two homozygous truncating KIF26A variants were associated with megacolon resembling Hirschsprung's disease without aganglionosis.^5^ In our study, we provide the first intestinal pathology study showing segmental aganglionosis in myenteric and submucosal plexuses in a KIF26A patient. These findings support the PIPO‐KIF26A association and suggest that KIF26A deficiency should be considered in the differential diagnosis of ENS disorders regardless of the presence of aganglionosis.^5^

In our cohort, we observed significant cortical malformations, including polymicrogyria, heterotopia, gyration anomalies, hydrocephalus, corpus callosum hypoplasia, and septum pellucidum agenesis, which are core neuroradiologic features of KIF26A patients^5^ (Table 1). Interestingly, these findings overlap with those seen in patients with Rac‐Rho signaling disorders,^3^, ^8^, ^15^, ^20^ suggesting a crucial role for KIF26A in human cortical development. Additionally, some brain defects identified in our patients, such as cerebellar foliar anomalies and hippocampal incomplete rotation, have never been reported and expand the neuroimaging spectrum of KIF26A‐related disorder.^5^

In conclusion, we describe three novel, patients with *KIF26A*‐related disorder presenting with brain developmental defects and PIPO‐like features. Our findings expand the genotype and phenotype spectrum of this emerging condition, suggesting that PIPO can be part of the clinical spectrum. Further reports will be crucial to dissect the molecular mechanisms and long‐term outcomes in this disorder.

## CONFLICT OF INTEREST STATEMENT

The authors declare no conflicts of interest.

### PEER REVIEW

The peer review history for this article is available at https://www.webofscience.com/api/gateway/wos/peer‐review/10.1111/cge.14621.

## Supporting information


**Data S1:** Supporting Information.

**Table S1.** Summary of genetic variants associated with Hirschsprung disease (HSCR) identified in the study cohort.

**Table S2:** Total energy scores for the best‐minimized structures of wild‐type and KIF26A variants.

## Data Availability

The data that support the findings of this study are available from the corresponding author upon reasonable request.
