# Supplementary material for: Novel KIF26A variants associated with pediatric intestinal pseudo‐obstruction (PIPO) and brain developmental defects
Source: Clin Genet. 2024 Sep 21;107(1):83–90. doi: 10.1111/cge.14621 (PMC11608842; doi:10.1111/cge.14621)
Supplement: Supplementary file 1 — Data S1: Supporting Information. [file CGE-107-83-s002.docx]

**Supplemental Material**

**1. Supplemental Methods**

**2. Supplemental Figures**

**3. Supplemental Tables**

**4. Supplemental References**

**1. 3D protein modeling Methods**

The wild-type (WT) protein comprises 1882 amino acids, and its structure has not been determined experimentally. To evaluate the structural impact of KIF26A missense variants, we used two approaches to capture both major and minor effects on protein structure. We employed AlphaFold3 to predict the structure of the mutated protein without using the wild-type protein as a template. This was done to better capture possible misfolding of proteins due to the variants and avoid bias in the predictions. Furthermore, This allowed us to assess the effects of the variants identified in the patients within the WT protein structure. We then minimized the best structures predicted by AlphaFold3 and those generated using YASARA by applying Rosetta 3.14 independently 10 times to each structure^SR1^. The minimization process involved initially constraining the protein's structure to its starting coordinates and gradually releasing these constraints, allowing the protein to adopt its most stable and energetically favorable conformation. Ultimately, we calculated the energy levels of the best-minimized structures using Rosetta 3.14 to provide insights into the stability of the mutated proteins. The 3D structures of the proteins were visualized using UCSF ChimeraX, as presented in Figures S1 to S4 ^SR,2,3,4,5,6^.

**2. Supplemental Figures**


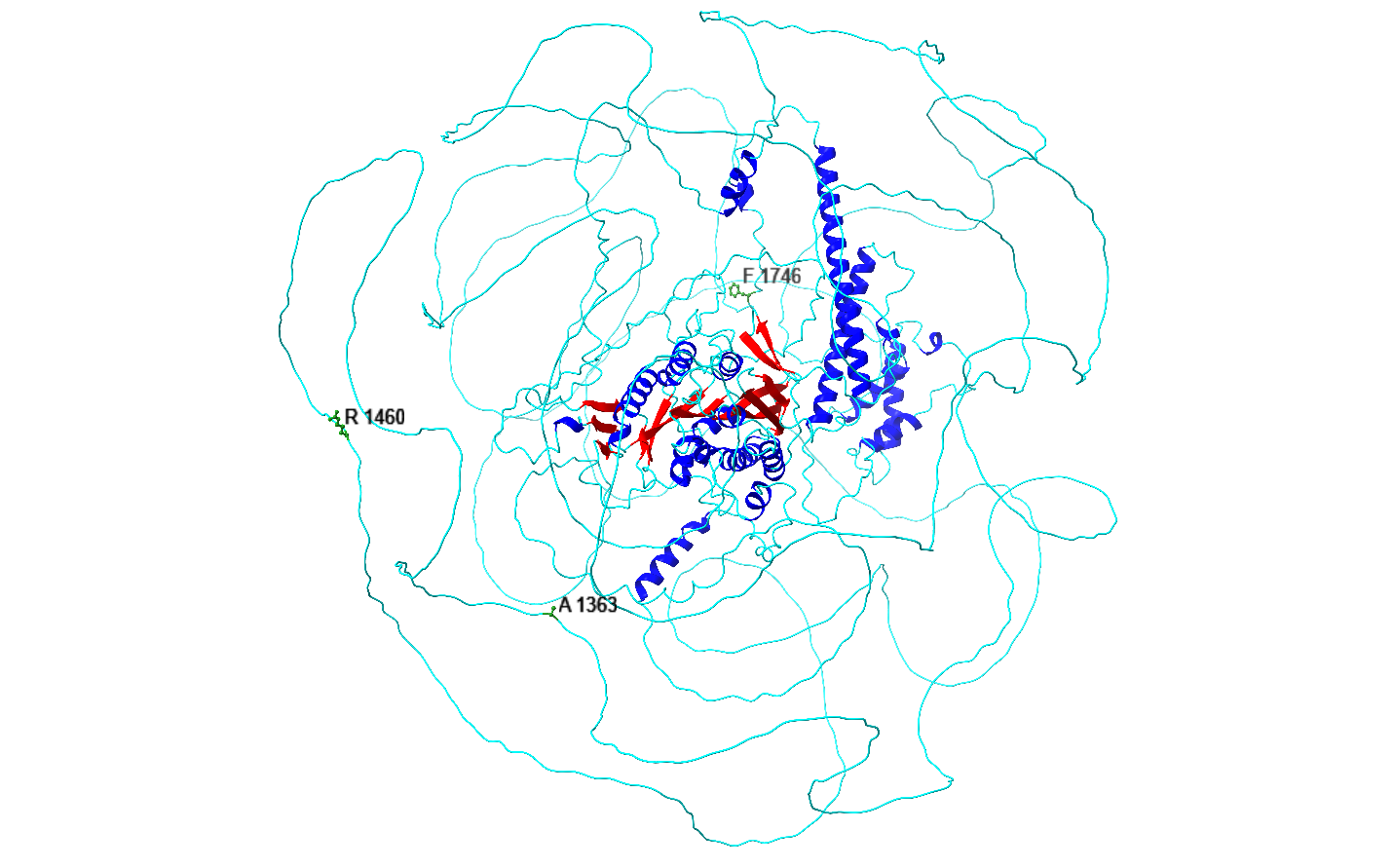


**Figure S1**: The predicted 3D structure of the wild-type Kinesin-like protein KIF26A, generated using AlphaFold3, visualized using UCSF ChimeraX, is shown. The amino acid residues corresponding to the identified variants are highlighted in forest green and displayed in ball-and-stick atom style with labels.

**
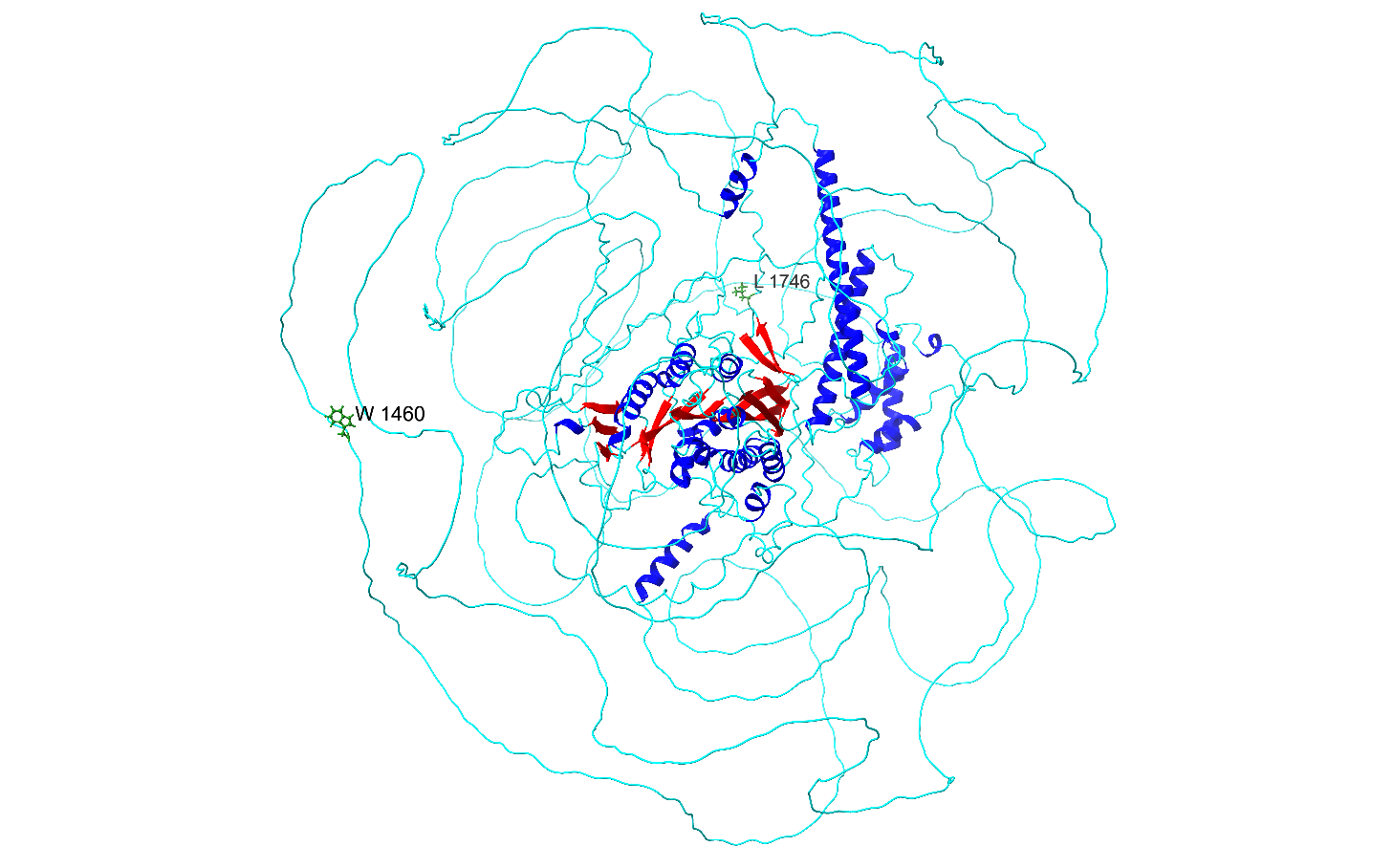
**

**Figure S2. Best-minimized structure of patient 1 variant (amino acid swap in wild-type)**


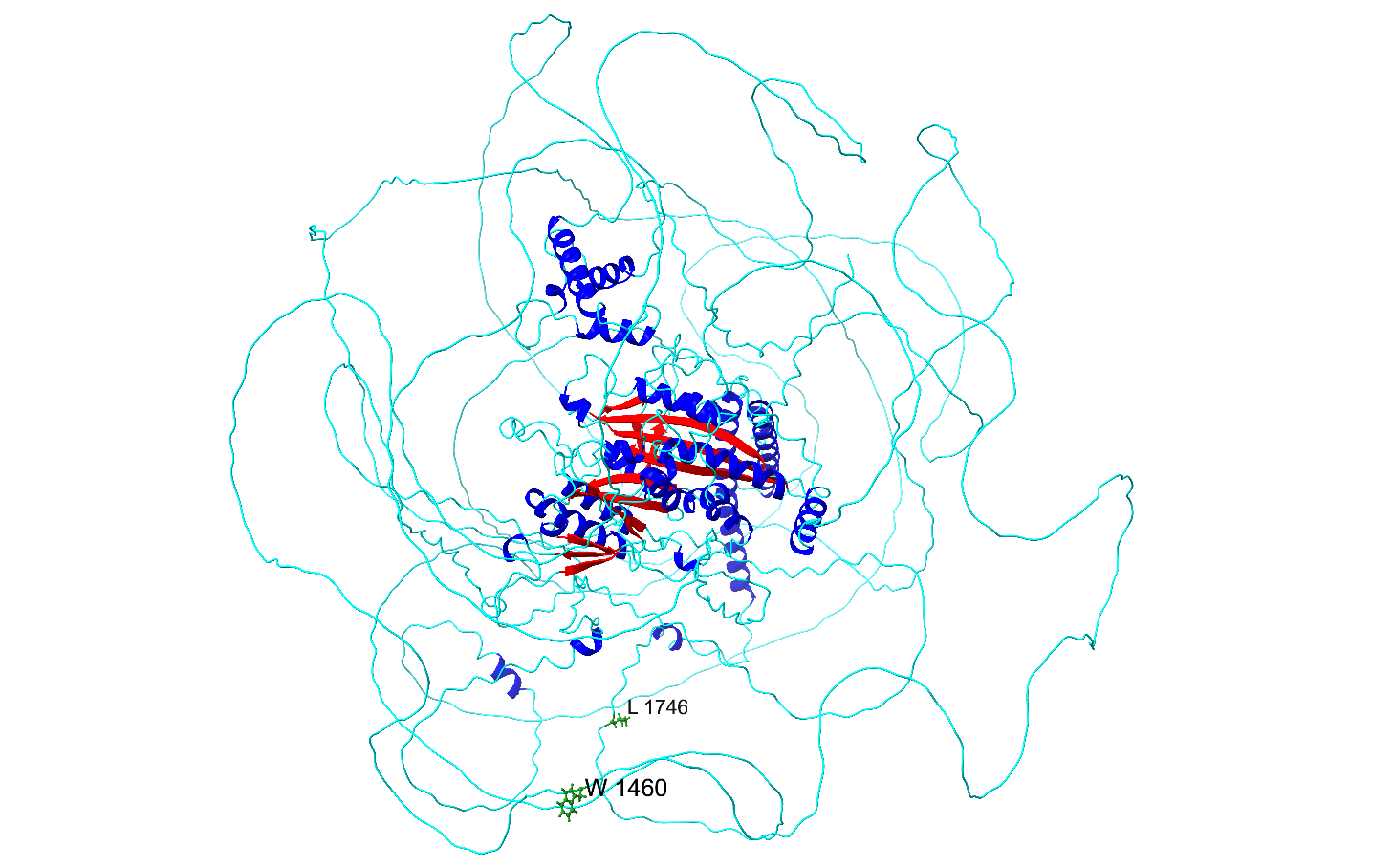


**Figure S3. Best-minimized structure of patient 1 variant (predicted by AlphaFold3, visualized by UCSF ChimeraX)**

**
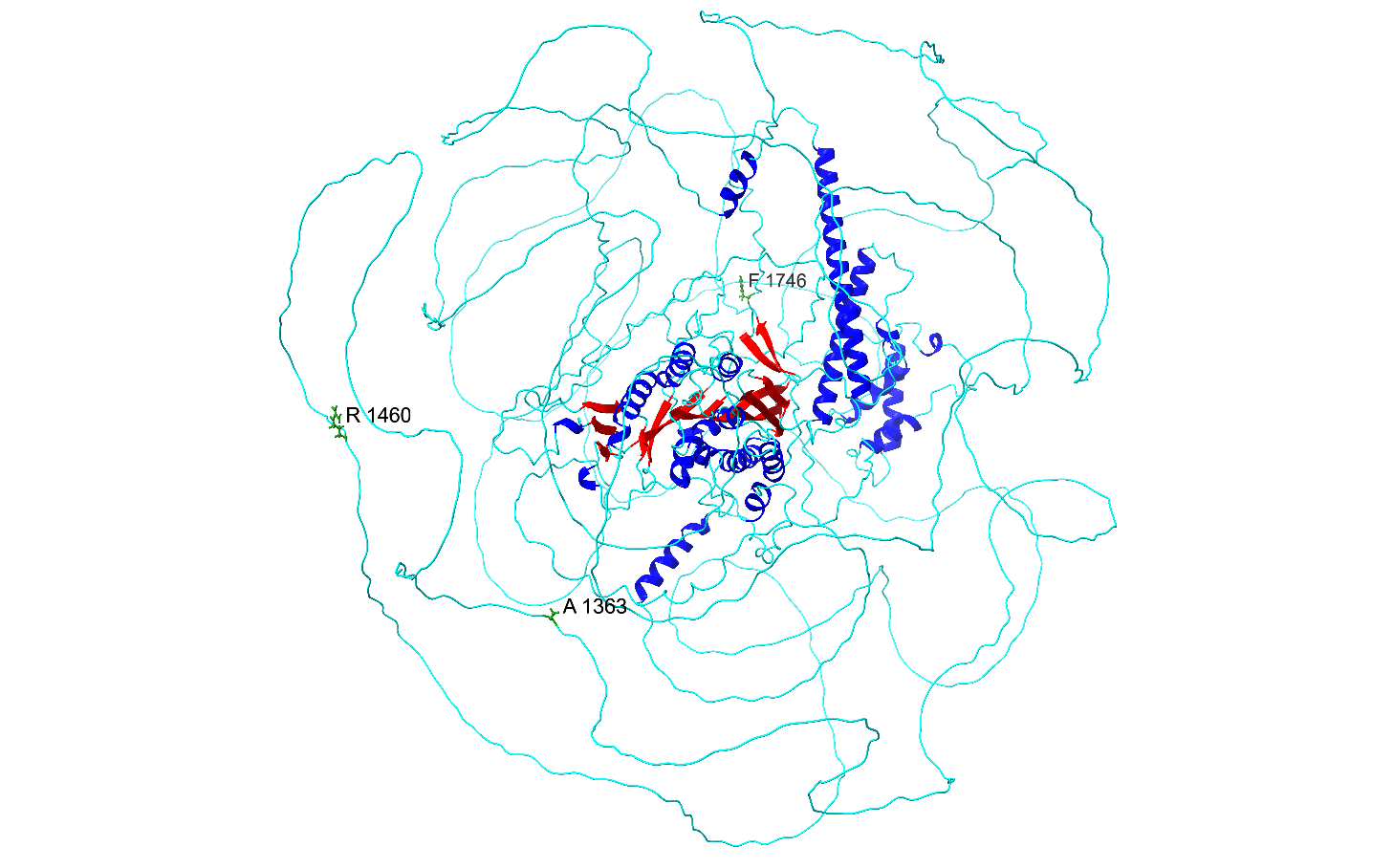
**

**Figure S4.** **Best-minimized structure of wild-type (WT) (predicted by AlphaFold3, visualized by UCSF ChimeraX)**

**3. Supplemental Tables**

**Table S1. Summary of genetic variants associated with Hirschsprung disease (HSCR) identified in the study cohort.**

This table lists all the genetic variants identified in the HSCR patients analyzed in the study. The columns provide detailed information about each variant, including the gene name, nucleotide change, amino acid change, inheritance pattern, and predicted pathogenicity. Variants are classified according to their pathogenicity as either benign, likely benign, uncertain significance, likely pathogenic, or pathogenic, based on ACMG guidelines. The frequency of each variant in the general population is also provided, as well as any previous literature references supporting their association with HSCR. Additionally, the table includes functional annotations and any known phenotypic correlations observed in patients carrying these variants.

**Table S2: Total energy scores for the best-minimized structures of wild-type and KIF26A variants**

**4. Supplemental References**

1. Abramson J, Adler J, Dunger J, et al. Accurate structure prediction of biomolecular interactions with AlphaFold 3. Nature. 2024;630:493-500. doi:10.1038/s41586-024-07487-w.
2. Krieger E, Vriend G. YASARA View—molecular graphics for all devices—from smartphones to workstations. Bioinformatics. 2014;30(20):2981-2982. doi:10.1093/bioinformatics/btu426.
3. Leaver-Fay A, Tyka M, Lewis SM, et al. Rosetta3: An Object-Oriented Software Suite for the Simulation and Design of Macromolecules. In: Johnson ML, Brand L, eds. Methods in Enzymology. Burlington, MA: Academic Press, 2011:545-574. doi:10.1016/B978-0-12-381270-4.00019-6.
4. Greer L. ASRM Guideline: Preimplantation Genetic Testing for Monogenic Disorders. ObG Proj. 2023 Mar;2024. Available from: https://www.obgproject.com/2023/06/23/asrm-guideline-preimplantation-genetic-testing-for-monogenic-disorders/.
5. Butler. Preimplantation Genetic Testing (PGT). Fertility Women & Infants. 2023 Mar;2024. Available from: <https://fertility.womenandinfants.org/treatment/preimplantation-genetic-testing>.
6. Pettersen EF, Goddard TD, Huang CC, et al. UCSF ChimeraX: Structure visualization for researchers, educators, and developers. *Protein Sci.* 2021 Jan;30(1):70-82. doi:10.1002/pro.3943. Epub 2020 Oct 22. PMID: 32881101; PMCID: PMC7737788.
